# Supplementary material for: Whole-genome sequencing reveals inter-household networks of gut-colonising ESBL-producing Escherichia coli in two rural Malawian districts
Source: Nat Commun. 2026 Oct 14;17:11612. doi: 10.1038/s41467-026-78449-1 (PMC13755399; doi:10.1038/s41467-026-78449-1)

Whole-genome sequencing reveals inter-household networks of gut-colonising ESBL-producing *Escherichia coli* in two rural Malawian districts

Angus M. O'Ferrall, David Lally, Peter Makaula, Gladys Namacha, Joseph M. Lewis, Patrick Musicha, Richard N. Goodman, Ellie Allman, Sabrina Moyo, Claire S. Waddington, Sekeleghe A. Kayuni, Nicholas A. Feasey, Janelisa Musaya, J. Russell Stothard, Adam P. Roberts

Supplementary information:

Supplementary Table 1. Logistic regression model exploring participant characteristics associated with carriage of ESBL-Ec strains detected in multiple households.

Supplementary Table 2. ARG co-occurrence and virulence in medoid genomes of ESBL-Ec strains identified in ≥3 households.

Supplementary Table 3. Comparison of medoid isolate selection using ANI-based versus core genome SNP-based distance matrices within ESBL-Ec strains identified in ≥3 households.

Supplementary Figure 1. ESBL-Ec gut colonisation groups after baseline and 12-month follow-up sampling.

Supplementary Figure 2. ANI hierarchical clustering dendrogram.

Supplementary Figure 3. Plasmid incompatibility groups among ESBL-Ec isolates (n=159).

Supplementary Figure 4. ARG profile dynamics among ESBL-Ec isolates (2023–24).

Supplementary Figure 5. ST profile dynamics among ESBL-Ec isolates (2023–24).

Supplementary Figure 6. BLAST ring alignments and associated gene cluster comparisons of plasmids sharing the same ESBL gene and incompatibility group from ESBL-Ec strains identified in ≥3 households.

Supplementary Figure 7. Yersiniabactin (ybt) biosynthetic gene cluster comparison.

Supplementary Figure 8. Scatter plot of pairwise SNP distances between same-strain isolates identified in ≥3 households.

Supplementary Table 1. Logistic regression model exploring participant characteristics associated with carriage of ESBL-Ec strains detected in multiple households. Outcome variable = carriage of a strain identified in ≥2 households at any point during the study. Participants providing a follow-up sample had increased opportunity for detection across two sampling rounds; follow-up status was therefore included as an adjustment variable rather than interpreted as an exposure. All reported *P*-values are two-sided and were derived from Wald tests within the generalised linear model framework.

Predictor variable		Adjusted odds ratio (aOR)	95% CI	*P*-value
Age (per year) at baseline		0.99	0.96–1.02	0.464
Sex	Female	1	—	—
	Male	0.71	0.39–1.28	0.258
District	Nsanje	1	—	—
	Mangochi	0.96	0.47–1.97	0.920
Follow-up sample provided	No	1	—	—
	Yes	2.23	1.17–4.39	0.017

Supplementary Table 2. ARG co-occurrence and virulence in medoid genomes of ESBL-Ec strains identified in ≥3 households. For each strain, the table reports the representative isolate, the ESBL gene detected and its genomic context, ARGs located on the same replicon as the ESBL gene and ARGs located on other replicons. Extraintestinal pathogenic *E. coli* (ExPEC) virulence categories (A = adhesin, IU = iron uptake, P = protectin, T = toxin), presence or absence of the yersiniabactin biosynthetic gene cluster (*ybt* BGC) and diarrhoeagenic pathotype (EPEC = enteropathogenic *E. coli*) are also shown.

Phylogroup	Strain (ST)	Isolate (GenBank accession)	ESBL gene (associated replicon type)	ARGs on replicon with ESBL gene	ARGs on other replicons	ExPEC virulence categories	*ybt* BGC	Diarrhoeagenic pathotype
A	i (ST206)	cM2a (JBYTPB000000000)	$bla_{\text{CTX-M-27}}$ (IncFIB plasmid)	*aac(6')-Ib-cr, aadA5, aadA16, ARR-3, dfrA17, dfrA27, floR, qnrB6, sul1, sul2, tet(A)*	-	A, P	No	-
	ii (ST10)	cM8a (JBYTPC000000000)	$bla_{\text{CTX-M-15}}$ (IncB/O/K/Z plasmid)	*dfrA1, qnrS1*	*aph(6)-Id, aph(3'')-Ib, sul2, tet(A)*	A, IU, P, T	Yes	-
	iii (ST8025)	fS149a (JBYTOO000000000)	$bla_{\text{CTX-M-15}}$ (chromosome)	*mph(A), tet(B)*	*aph(3'')-Ib, aph(6)-Id,* $bla_{\text{TEM-1B}}$*, dfrA8, mph(A), sul2*	A, IU, P	Yes	-
	iv (ST44)	cS76a (JBYTOX000000000)	$bla_{\text{CTX-M-15}}$ (IncFIA/IncFIB plasmid)	*aadA5, dfrA17, mph(A), sul1, tet(B)*	*aph(3'')-Ib, aph(6)-Id, sul2*	A, IU, P	Yes	-
	v (ST13823)	fS25a (JBYTOS000000000)	$bla_{\text{CTX-M-15}}$ (IncFIB plasmid)	*aadA5, dfrA17, qnrS1, sul1*	*aph(3'')-Ib, aph(6)-Id, sul2*	A, P	No	EPEC
	vi (ST10)	cS100a (JBYTOV000000000)	$bla_{\text{CTX-M-15}}$ (chromosome)	-	*aac(3)-IId,* $bla_{\text{TEM-1B}}$	A, IU, P	Yes	-
B1	vii (ST3580)	cS108a (JBYTOU000000000)	$bla_{\text{CTX-M-15}}$ (chromosome)	*qnrS1*	*aph(3'')-Ib, aph(6)-Id,* $bla_{\text{TEM-1B}}$*, dfrA8, sul2*	A, P	No	-
	viii (ST1015)	cS33a (JBYTOZ000000000)	$bla_{\text{CTX-M-15}}$ (IncY plasmid)	*aph(3'')-Ib, aph(6)-Id,* $bla_{\text{TEM-1B}}$*, dfrA14, qnrS1, sul2*	*tet(B)*	P	No	-
	ix (ST58)	fS54a (JBYTOQ000000000)	$bla_{\text{CTX-M-27}}$ (IncFIA/IncFIB plasmid)	*aadA5, aph(3'')-Ib, aph(6)-Id, dfrA17, mph(A), sul1, sul2, tet(A)*	-	A, IU, P	No	-
	x (ST2852)	fS58a (JBYTOP000000000)	$bla_{\text{CTX-M-15}}$ (IncY plasmid)	*aph(3'')-Ib, aph(6)-Id,* $bla_{\text{TEM-1B}}$*, dfrA14, qnrS1, sul2, tet(A)*	$bla_{\text{TEM-1B}}$*, mph(A)*	A, P	No	-
	xi (ST1015)	fM51a (JBYTOT000000000)	$bla_{\text{CTX-M-15}}$ (IncY plasmid)	*aph(3'')-Ib, aph(6)-Id,* $bla_{\text{TEM-1B}}$*, dfrA14, qnrS1, sul2*	*tet(B)*	P	No	-
B2	xii (ST131)	cS53a (JBYTPA000000000)	$bla_{\text{CTX-M-15}}$ (chromosome)	*aac(6')-Ib-cr,* $bla_{\text{OXA-1}}$	*aac(3)-IId, aadA5, aph(3'')-Ib, aph(6)-Id,* $bla_{\text{TEM-1B}}$*, dfrA17, mph(A), sul1, sul2, tet(A)*	A, IU, P, T	Yes	-
	xiii (ST131)	cS91a (JBYTOW000000000)	$bla_{\text{CTX-M-15}}$ (chromosome)	-	*aadA5, aph(3'')-Ib, aph(6)-Id,* $bla_{\text{TEM-141}}$*, dfrA17, mph(A), sul1, sul2, tet(A)*	A, IU, P, T	No	-
D	xiv (ST38)	cS51a (JBYTOY000000000)	$bla_{\text{CTX-M-27}}$ (IncFIB/IncFII plasmid)	*aadA5, aph(3'')-Ib, aph(6)-Id, dfrA17, mph(A), sul1, sul2, tet(A)*	-	A, IU, P	Yes	-
	xv (ST38)	fS10a (JBYTOR000000000)	$bla_{\text{CTX-M-15}}$ (chromosome)	*ant(3'')-Ia, aph(3'')-Ib, aph(6)-Id,* $bla_{\text{TEM-1B}}$*, dfrA1, sul2, tet(D)*	-	A, IU, P	Yes	-

Supplementary Table 3. Comparison of medoid isolate selection using ANI-based versus core genome SNP-based distance matrices within ESBL-Ec strains identified in ≥3 households. The core genome SNP matrix was generated using the *E. coli* K-12 reference genome as reference. The chromosomal SNP distances between medoids were generated using the chromosomal sequences of hybrid-assembled ANI-based medoids as references.

Strain (ST)	ANI-based medoid	SNP-based medoid (K-12 core genome mapping)	Same/different	Chromosomal SNPs between medoids (strain-specific mapping)
i (ST206)	cM2a	cM2a	Same	0
ii (ST10)	cM8a	cM9b	Different	0
iii (ST8025)	fS149a	fS149a	Same	0
iv (ST44)	cS76a	cS68a	Different	3
v (ST13823)	fS25a	fS95a	Different	3
vi (ST10)	cS100a	cS100a	Same	0
vii (ST3580)	cS108a	cS164a	Different	2
viii (ST1015)	cS33a	cS30a	Different	0
ix (ST58)	fS54a	fS191a	Different	2
x (ST2852)	fS58a	fS58a	Same	0
xi (ST1015)	fM51a	fM19a	Different	1
xii (ST131)	cS53a	cS61a	Different	1
xiii (ST131)	cS91a	cS80a	Different	1
xiv (ST38)	cS51a	cS132a	Different	7
xv (ST38)	fS10a	fS63b	Different	1

Supplementary Figure 1. ESBL-Ec gut colonisation groups after baseline and 12-month follow-up sampling. Only data from participants providing samples at baseline and follow-up are shown.

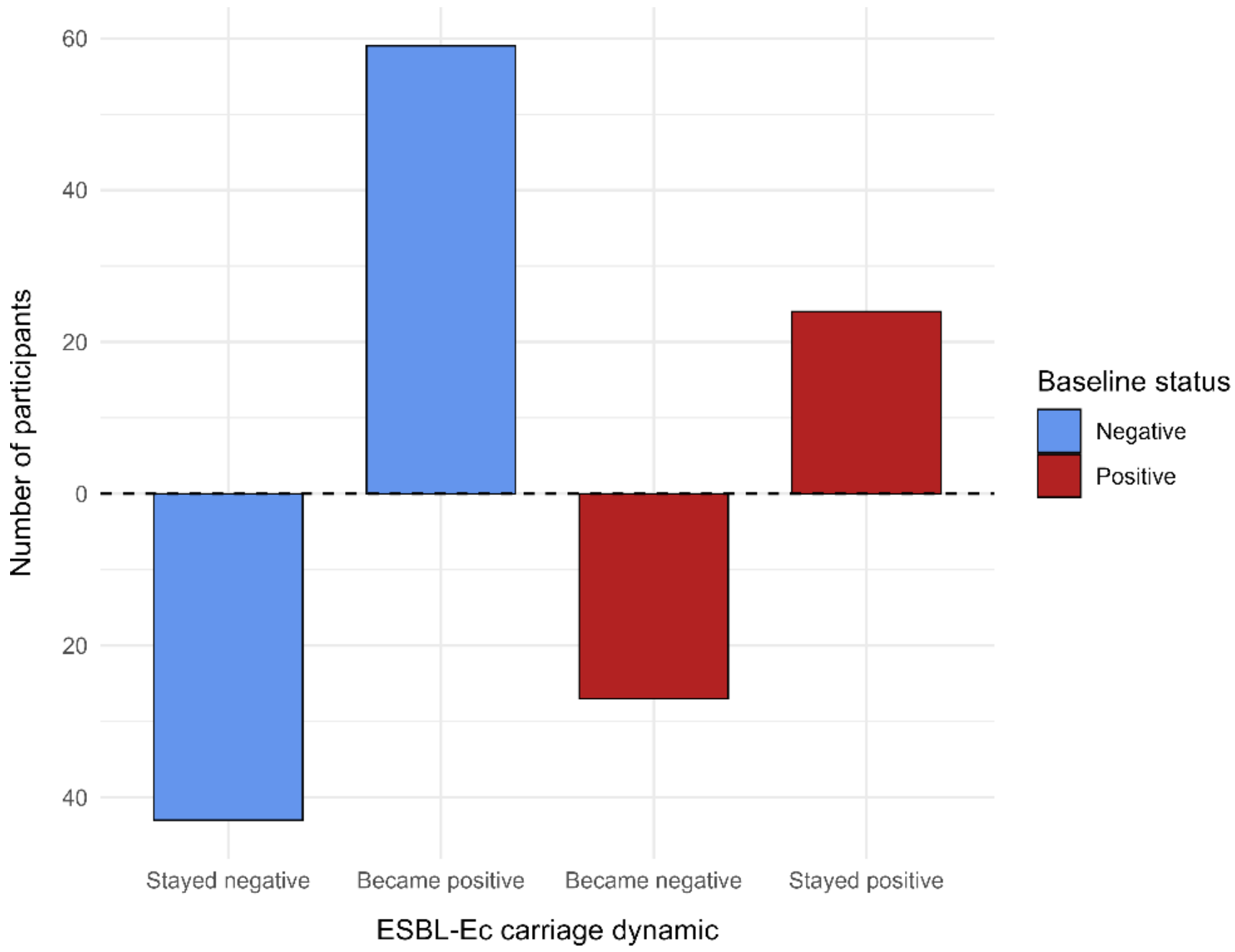

Supplementary Figure 2. ANI hierarchical clustering dendrogram. Hierarchical clustering of pairwise ANI distances using average linkage was used to identify strains. The dashed red line indicates the distance threshold used to define same-strain genomes (0.0001; equivalent to 99.99% ANI). Genomes clustering below this threshold were classified as belonging to the same strain.

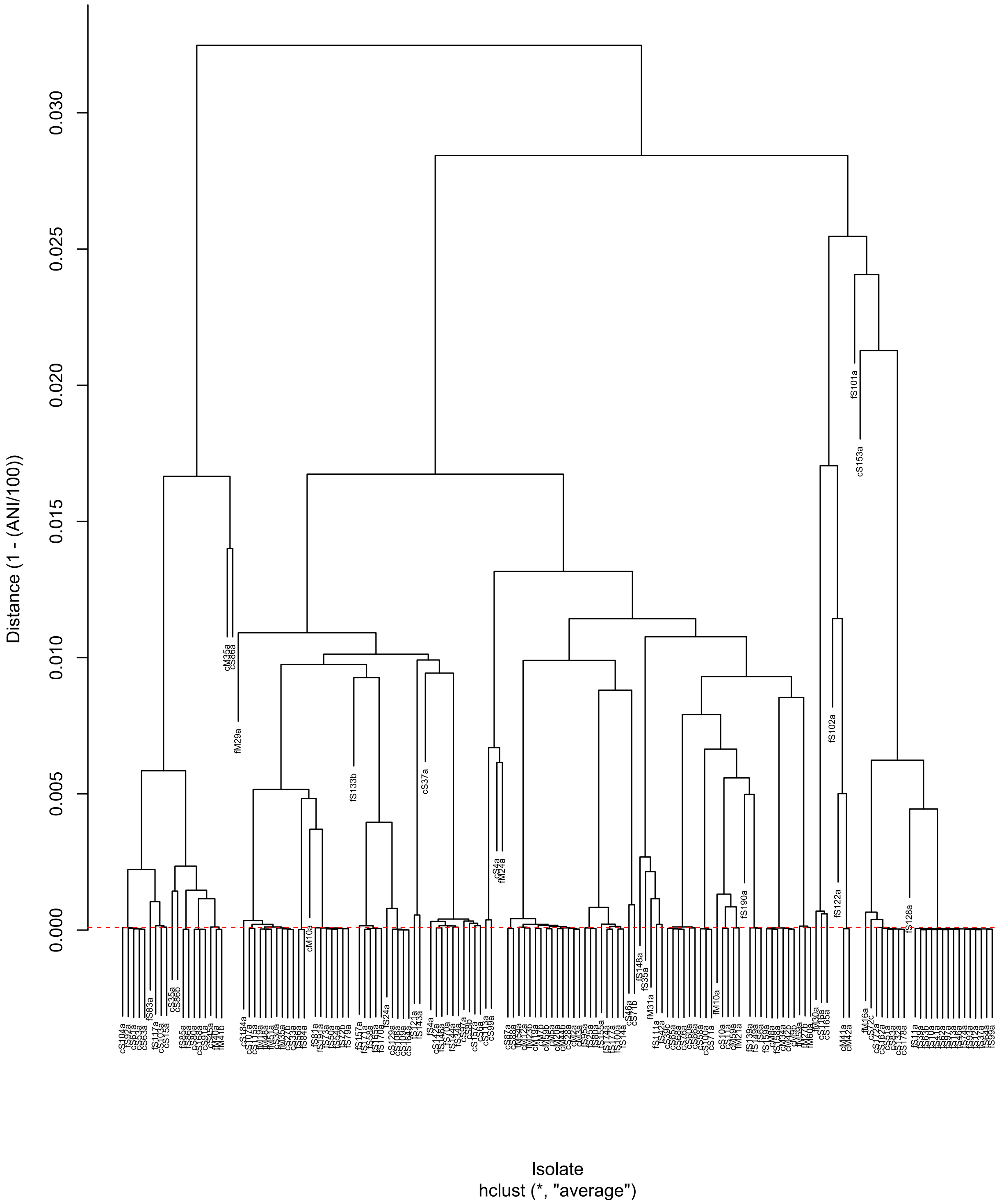

Supplementary Figure 3. Plasmid incompatibility groups identified within ESBL-Ec isolates (n=159).

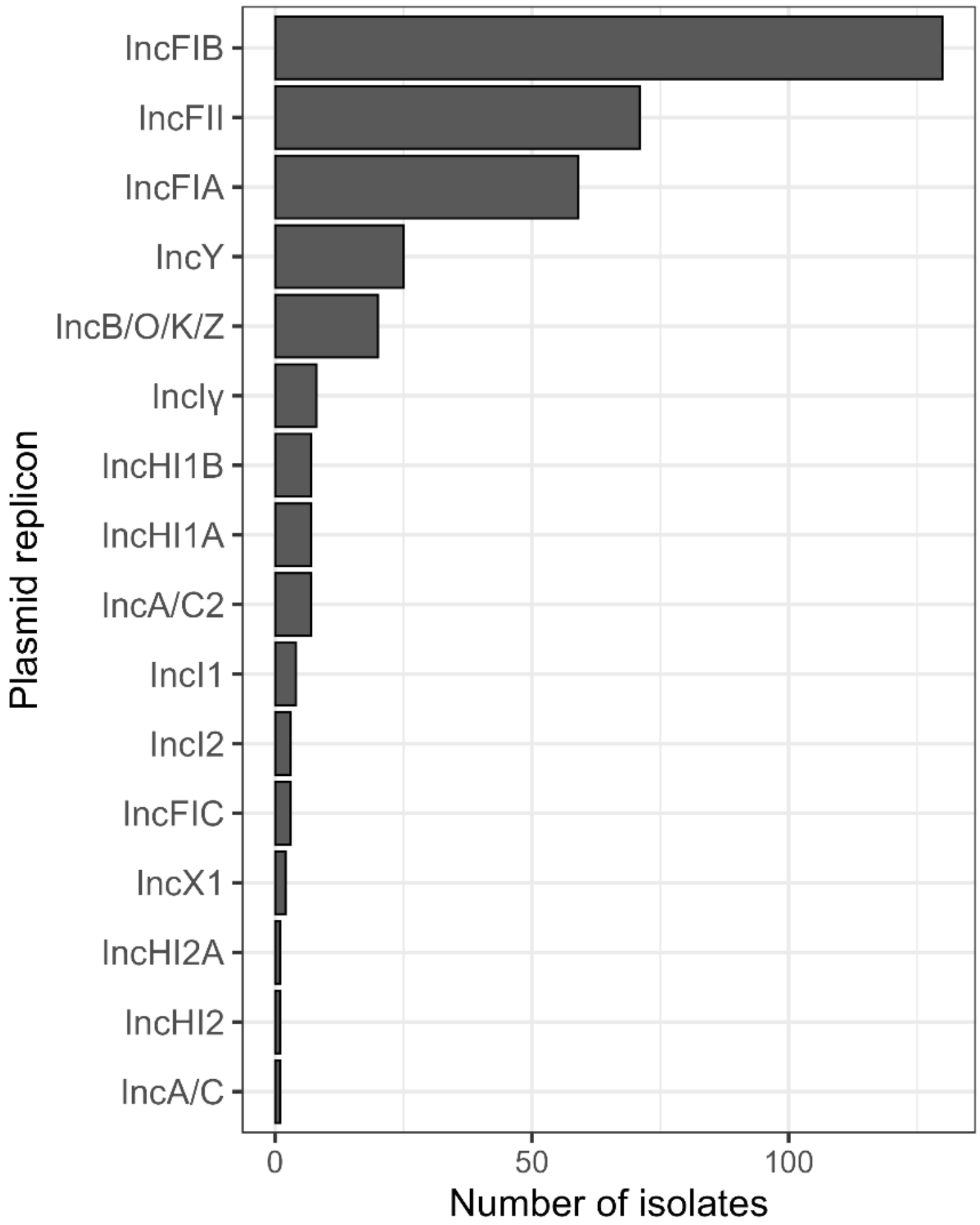

Supplementary Figure 4. ARG profile dynamics among ESBL-Ec isolates (2023–24). The percentage of isolates containing each AMR determinant is shown for 2023 (n=76) and 2024 (n=83).

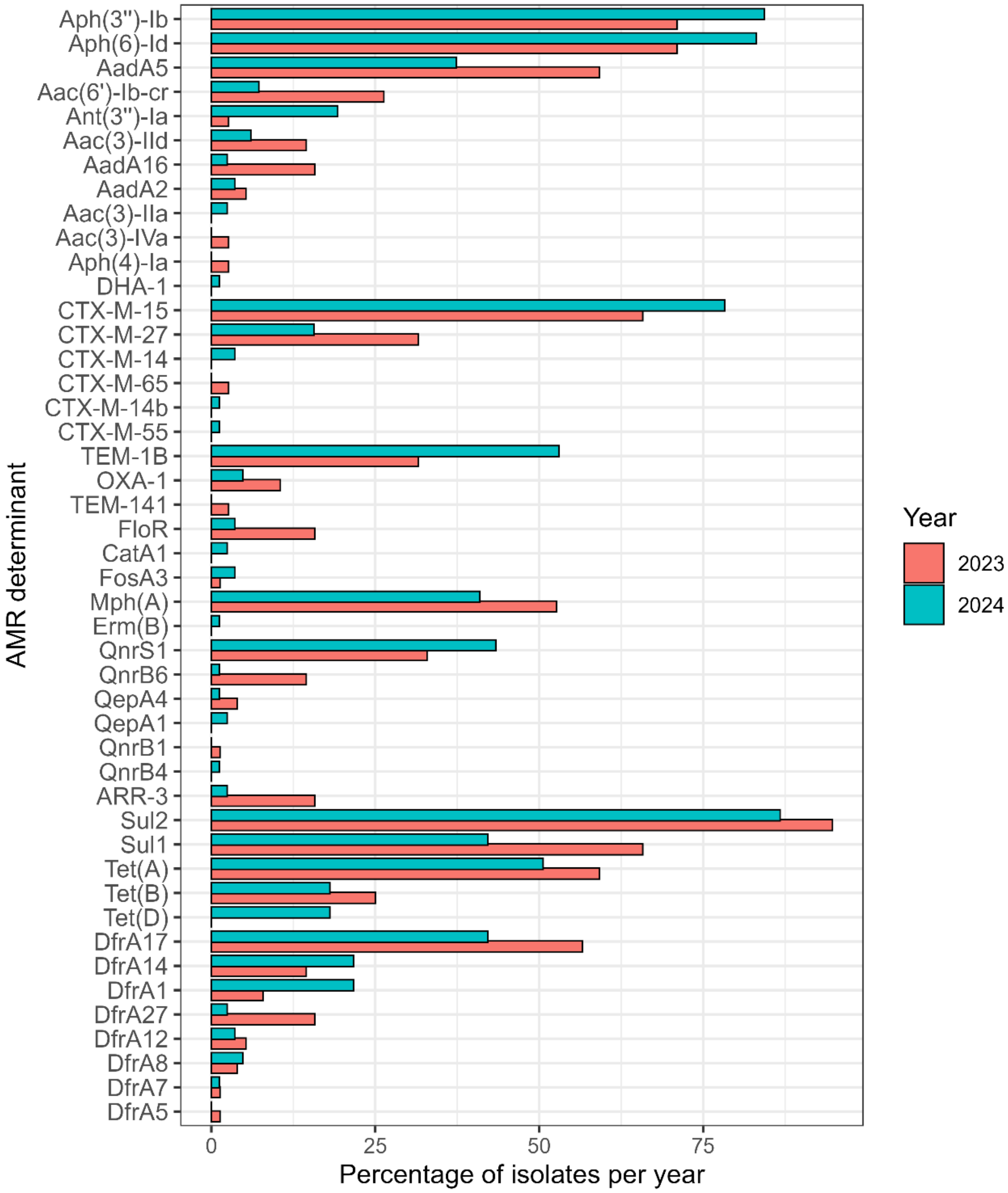

Supplementary Figure 5. ST profile dynamics among ESBL-Ec isolates (2023–24). The percentage of isolates from each ST is shown for 2023 (n=76) and 2024 (n=83).

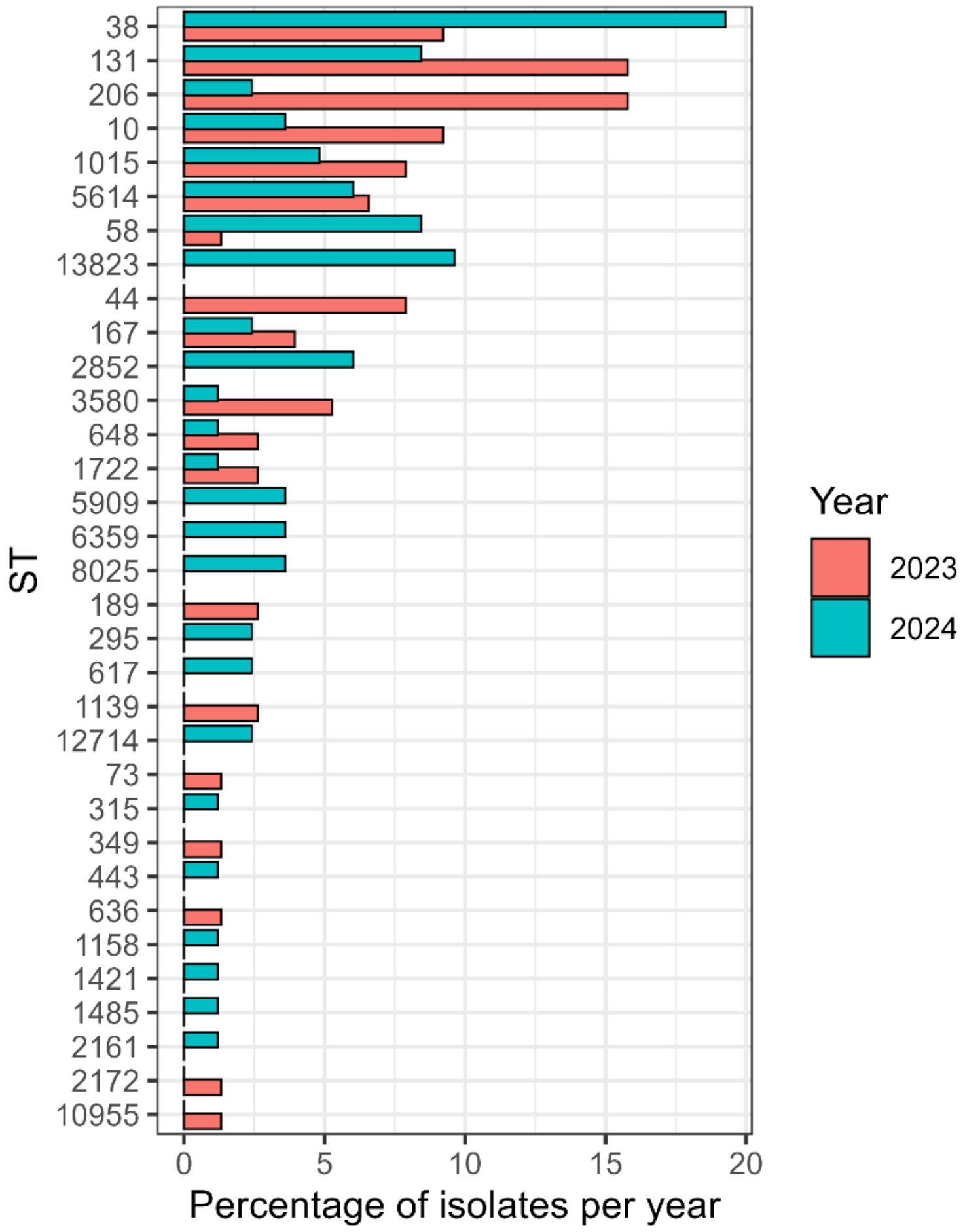

Supplementary Figure 6. BLAST ring alignments and associated gene cluster comparisons of plasmids sharing the same ESBL gene and incompatibility group from ESBL-Ec strains identified in ≥3 households. BLAST ring alignments show regions of minimum 90% nucleotide ID over a minimum of 1 kbp. For comparisons of >2 plasmids with the same ESBL gene and incompatibility group, the plasmid with the greatest mean number of aligned bases relative to others in the group was chosen as the reference. Regions coding for hypothetical proteins are not displayed.

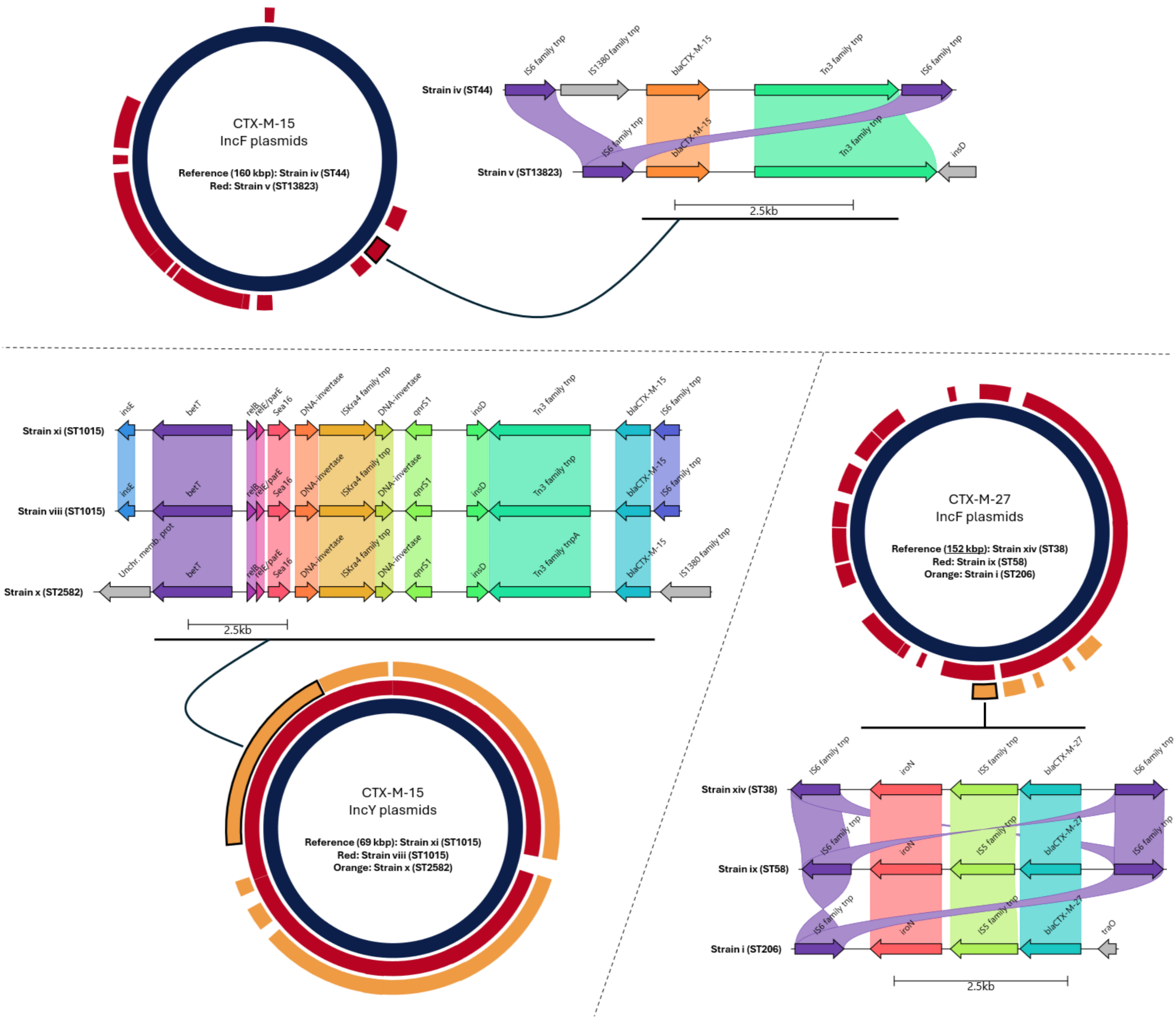

Supplementary Figure 7. Yersiniabactin (*ybt*) biosynthetic gene cluster comparison. Annotated medoid genomes of strains found in ≥3 households containing the *ybt* biosynthetic gene cluster are included. All links share 100% nucleotide identity. An IS*4* family transposase inserted between the *irp2* and *ybtA* genes in cM8a.

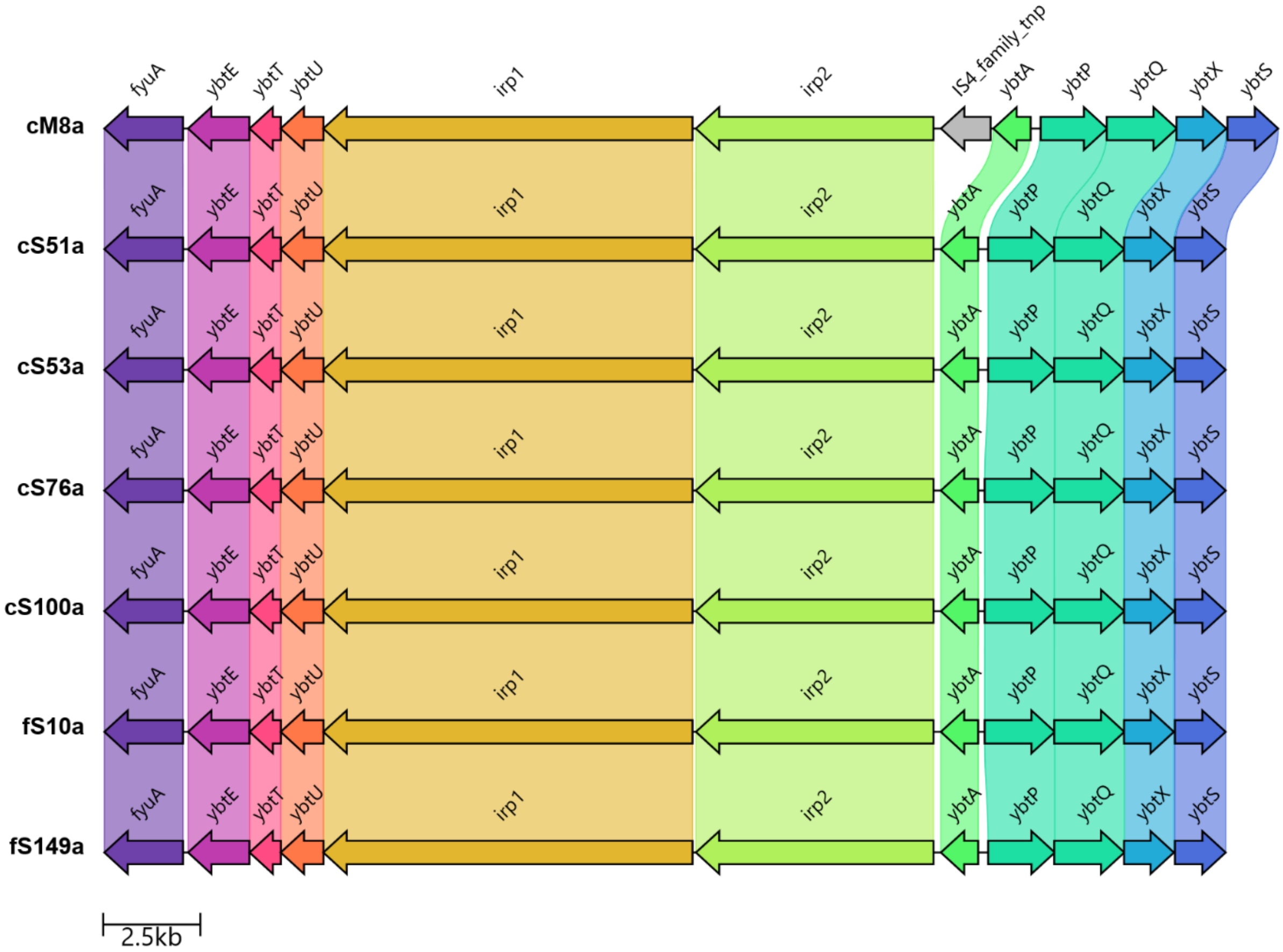

Supplementary Figure 8. Scatter plot of pairwise SNP distances between same-strain isolates identified in ≥3 households. Pairwise comparisons (n=190) are shown between isolates sampled concurrently from different households in the same village.

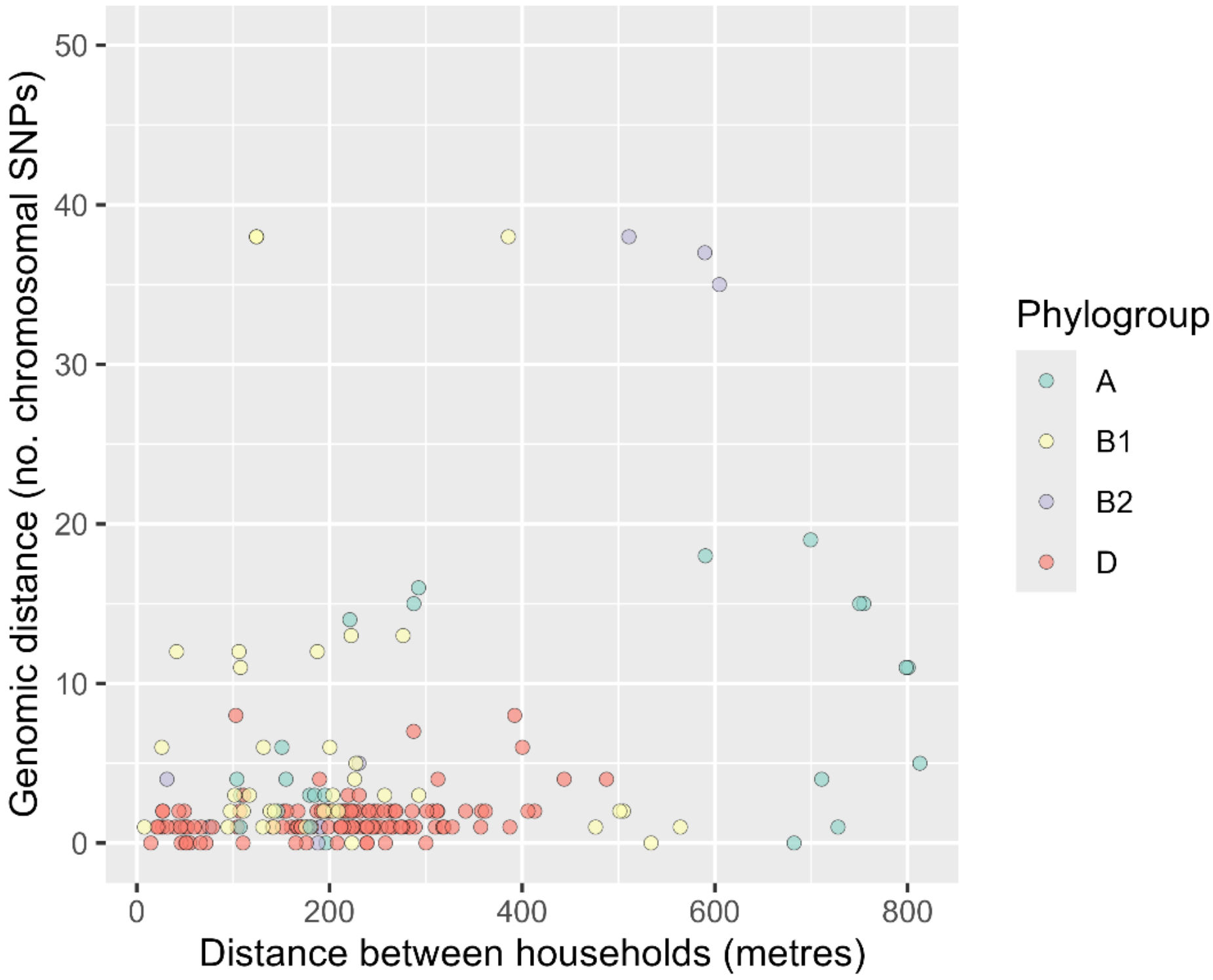

Supplement: Supplementary file 1 [file 41467_2026_78449_MOESM1_ESM.pdf]
