# Supplementary material for: Whole-genome sequencing reveals inter-household networks of gut-colonising ESBL-producing Escherichia coli in two rural Malawian districts
Source: Nat Commun. 2026 Oct 14;17:11612. doi: 10.1038/s41467-026-78449-1 (PMC13755399; doi:10.1038/s41467-026-78449-1)
Supplement: Supplementary file 2 [file 41467_2026_78449_MOESM2_ESM.pdf]

nature portfolio

Corresponding author(s): Adam P. Roberts

Last updated by author(s): Aug 21, 2026

Reporting Summary

Nature Portfolio wishes to improve the reproducibility of the work that we publish. This form provides structure for consistency and transparency in reporting. For further information on Nature Portfolio policies, see our Editorial Policies and the Editorial Policy Checklist.

Statistics

For all statistical analyses, confirm that the following items are present in the figure legend, table legend, main text, or Methods section.

n/a	Confirmed	
☐	☒	The exact sample size (n) for each experimental group/condition, given as a discrete number and unit of measurement
☐	☒	A statement on whether measurements were taken from distinct samples or whether the same sample was measured repeatedly
☐	☒	The statistical test(s) used AND whether they are one- or two-sided<br>*Only common tests should be described solely by name; describe more complex techniques in the Methods section.*
☐	☒	A description of all covariates tested
☐	☒	A description of any assumptions or corrections, such as tests of normality and adjustment for multiple comparisons
☐	☒	A full description of the statistical parameters including central tendency (e.g. means) or other basic estimates (e.g. regression coefficient) AND variation (e.g. standard deviation) or associated estimates of uncertainty (e.g. confidence intervals)
☐	☒	For null hypothesis testing, the test statistic (e.g. F, t, r) with confidence intervals, effect sizes, degrees of freedom and P value noted<br>*Give P values as exact values whenever suitable.*
☒	☐	For Bayesian analysis, information on the choice of priors and Markov chain Monte Carlo settings
☒	☐	For hierarchical and complex designs, identification of the appropriate level for tests and full reporting of outcomes
☐	☒	Estimates of effect sizes (e.g. Cohen's d, Pearson's r), indicating how they were calculated

Our web collection on statistics for biologists contains articles on many of the points above.

Software and code

Policy information about availability of computer code

Data collection	The study used a questionnaire, which was programmed in electronic tablets (Samsung Electronics Co., Ltd.) using Kobo Toolbox (version 2022.2.3).
Data analysis	The following software packages were used (in alphabetical order):<br><br>ABRicate - Version 1.0.1<br>ape - Version 5.8.1<br>Autocycler - Version 0.5.2<br>Bakta - Version 1.10.4<br>CheckM - Version 1.1.2<br>ComplexHeatmap - Version 2.26.1<br>Dnaapler - Version 1.3.0<br>fastANI - Version 1.3.4<br>FastQC - Version 0.11.9<br>Filtlong - Version 0.2.1<br>FSA - Version 0.10.1<br>geosphere - Version 1.5.20<br>ggraph - Version 2.2.2<br>ggplot2 - Version 4.0.1<br>ggpubr - Version 0.6.2<br>ggspatial - Version 1.1.10

ggtree - Version 4.0.4
ggtreeExtra - Version 1.20.1
Gubbins - Version 2.3.4
igraph - Version 2.2.1
IQ-TREE - Version 1.6.1
Medaka - Version 2.0.1
MLST - Version 2.23.0
Panaroo - Version 1.5.2
phangorn - Version 2.12.1
Polypolish - Version 0.5.0
popPUNK - Version 2.7.5
Pypolca - Version 0.3.1
QUAST - Version 5.0.2
R - Version 4.5.2
SeqKit - Version 2.1.0
Shovill - Version 1.0.4
snp-dists - Version 0.8.2
SNP-sites - Version 2.5.1
Snippy - Version 4.6.0
SPAdes - Version 3.13.0
Trimmomatic - Version 0.39

For manuscripts utilizing custom algorithms or software that are central to the research but not yet described in published literature, software must be made available to editors and reviewers. We strongly encourage code deposition in a community repository (e.g. GitHub). See the Nature Portfolio guidelines for submitting code & software for further information.

Data

Policy information about availability of data

All manuscripts must include a data availability statement. This statement should provide the following information, where applicable:

- Accession codes, unique identifiers, or web links for publicly available datasets
- A description of any restrictions on data availability
- For clinical datasets or third party data, please ensure that the statement adheres to our policy

Data and code used to carry out analyses in R are available at Github (https://github.com/amoreo71/malawi_esbl_e_coli_genomics) and archived at Zenodo (https://zenodo.org/records/22000727). Reads from isolates sequenced in this study have been deposited in the Sequence Read Archive under BioProject PRJNA1399404 (https://www.ncbi.nlm.nih.gov/bioproject/PRJNA1399404/). Accession numbers are SRR36707243–SRR36707401 for short-reads and SRR36746900–SRR36746914 for long-reads. Accession numbers are linked to isolate identifiers in the Source Data file. Previously published genomic data used in comparative genomic analyses are available from the European Nucleotide Archive under the following projects: PRJEB26677 (https://www.ebi.ac.uk/ena/browser/view/PRJEB26677), PRJEB28522 (https://www.ebi.ac.uk/ena/browser/view/PRJEB28522), PRJEB37378 (https://www.ebi.ac.uk/ena/browser/view/PRJEB37378). Accession numbers for individual isolates from these datasets are provided in the archived Zenodo dataset.

Research involving human participants, their data, or biological material

Policy information about studies with human participants or human data. See also policy information about sex, gender (identity/presentation), and sexual orientation and race, ethnicity and racism.

Reporting on sex and gender	Sex was self-reported and was not specifically incorporated into the study design but was included in analyses. The distribution of participants by sex is reported in Table 1 of the manuscript.
Reporting on race, ethnicity, or other socially relevant groupings	Data on race, ethnicity and other socially relevant grouping were not reported in this study.
Population characteristics	Population characteristics are included in Table 1 of the manuscript.
Recruitment	This study was embedded in a pre-existing longitudinal freshwater parasite surveillance study (Hybridisation in Urogenital Schistosomiasis; HUGS) in rural southern Malawi. Participants aged 2 years or older who provided faecal samples as part of the HUGS study in June-July 2023 were eligible for inclusion in this AMR study, provided they were not acutely unwell at the time of sampling and gave additional informed consent for their samples to undergo microbiological analysis. Participants under the age of 16 provided assent alongside consent from a parent or guardian. Participants were not aware of their ESBL-producing E. coli colonisation status prior to recruitment and were recruited from an existing surveillance cohort rather than on the basis of infection status, thus the potential for self-selection bias was limited.
Ethics oversight	This study was conducted in accordance with all relevant ethical regulations. Ethical approvals were granted by The College of Medicine Research Ethics Committee, Malawi (approval nos. P.08/21/3381 and P.06/23/4135) and the Liverpool School of Tropical Medicine Research Ethics Committee, United Kingdom (approval no. 22-028). Written informed consent was obtained from all adult participants (aged ≥18 years) and from the parents or guardians of participants aged <18 years. Written assent was additionally obtained from participants aged 7–17 years.

Note that full information on the approval of the study protocol must also be provided in the manuscript.

Field-specific reporting

Please select the one below that is the best fit for your research. If you are not sure, read the appropriate sections before making your selection.

☒ Life sciences ☐ Behavioural & social sciences ☐ Ecological, evolutionary & environmental sciences

For a reference copy of the document with all sections, see nature.com/documents/nr-reporting-summary-flat.pdf

Life sciences study design

All studies must disclose on these points even when the disclosure is negative.

Sample size	No statistical methods were used to predetermine sample size. This study was embedded within a pre-existing longitudinal schistosomiasis surveillance programme in southern Malawi. All faecal samples collected as part of that programme were eligible for inclusion in this study, provided participants gave additional informed consent for their samples to undergo microbiological analysis.
Data exclusions	All eligible samples were included in the analyses. No data were excluded.
Replication	Analyses of whole-genome sequencing data were performed using reproducible pipelines on multiple independent bacterial isolates collected from different participants, households and timepoints, as described in the Methods. All data, software versions and code have been made publicly available to enable full replication of the computational analyses.
Randomization	As this was an observational study, participants were not allocated to experimental groups.
Blinding	As this was an observational study, blinding was not applicable.

Reporting for specific materials, systems and methods

We require information from authors about some types of materials, experimental systems and methods used in many studies. Here, indicate whether each material, system or method listed is relevant to your study. If you are not sure if a list item applies to your research, read the appropriate section before selecting a response.

Materials & experimental systems

n/a	Involved in the study	
☒	☐	Antibodies
☒	☐	Eukaryotic cell lines
☒	☐	Palaeontology and archaeology
☒	☐	Animals and other organisms
☒	☐	Clinical data
☒	☐	Dual use research of concern
☒	☐	Plants

Methods

n/a	Involved in the study	
☒	☐	ChIP-seq
☒	☐	Flow cytometry
☒	☐	MRI-based neuroimaging
