# Supplementary material for: Whole-genome sequencing reveals inter-household networks of gut-colonising ESBL-producing Escherichia coli in two rural Malawian districts
Source: Nat Commun. 2026 Oct 14;17:11612. doi: 10.1038/s41467-026-78449-1 (PMC13755399; doi:10.1038/s41467-026-78449-1)
Supplement: Supplementary file 3 [file 41467_2026_78449_MOESM3_ESM.pdf]

nature portfolio

Peer Review File

Whole-genome sequencing reveals inter-household networks of gut-colonising ESBL-producing Escherichia coli in two rural Malawian districts

Corresponding Author: Professor Adam Roberts

This file contains all reviewer reports in order by version, followed by all author rebuttals in order by version.

Version 0:

Reviewer comments:

Reviewer #1

(Remarks to the Author)
General
The manuscript reads well, presents interesting data and the methodology is sound. I especially appreciate the deep dive into the transmission network analysis including the sensitivity analysis on recombination masking. However, I have two main concerns outlined below, as well as some other major and minor queries.

Major
General point 1: the discussion is missing a point in my opinion on the background prevalence of certain successful clones (such as ST10 and ST131). These lineages have extensive transmission among healthy carriers in other studies (esp. ST131). Seeing clusters ii and xii, I wonder whether these clusters represent clones normally present in the population, rather than strains being transmitted among the villages.
General point 2: Did you consider doing a risk factor analysis, to see whether any contextual data/metadata are associated with sharing a strain with another household? The manuscript is now quite ecological in nature, describing the flow of ESBL-Ec between households, but it in the current form it unfortunately offers little information which could be used to prevent ESBL-Ec transmission in a setting like this (mentioned as the first sentence in the discussion). If this was not feasible, it should at least be mentioned in the discussion.
L140: Seventy-six ESBL-Ec were collected at baseline, but based on a prevalence of 34.1% among 211 participants, a total of 72 ESBL-Ec-positive participants are expected. Were isolates deduplicated in any way?
L176-178: how does the number of virulence gene categories per strain compare to the number of virulence gene categories in strains that were detected only in a single household?
L402-407: does this finding on two ST38 strains sharing <= 10 SNPs not imply that the ANI threshold was either too strict or that ANI might be somewhat inaccurate in defining strains which are later used for transmission analysis? Does this influence the recommendations in the next paragraph? How were the differences in accessory genome assessed? And did other ANI calculation methods arrive at similar measures of relatedness? I could not find information on this “outlier” isolate in the Results.

Minor
L98 and throughout the rest of the manuscript: please rephrase “clonal complexes” as this is an ambiguous term in this setting. This could for example also be interpreted as a set of related sequence types (i.e. ST-complex).
L131-133: do you have additional data on the participants lost to follow-up? Did these differ from others based on the variables you collected?
L149: were you surprised by the absence of carbapenemases in the study? This could be put into context in the discussion.
L170-171: “we observed high levels of genome- and 171 replicon-level ARG co-occurrence (median ARG count per genome = 9)”. What is meant by this? That the genomes contain many ARGs?
L225: Do you have any additional information on the location of boreholes? Is the geographical distance a proxy measure for sharing of a borehole?
L401: please add “%” after 99.99
L574: why were these geographical distance thresholds selected?

(Remarks on code availability)
The code uploaded to Github is the html output of an RMarkdown file. Alongside this, all input contextual data/metadata has been uploaded to the repository. All figures and tables used in the manuscript can be generated by copy-pasting and running the code in the html file. Code for genomic analysis has not been uploaded, but this has also been covered in the Methods section.

Reviewer #2

(Remarks to the Author)
This is a well written and thorough paper, which employs a range of approaches for an in depth exploration ESBL E. coli, presented clearly and concisely such that I genuinely enjoyed reading this paper.

I had some suggestions, comments, and questions that I think may strengthen the paper and/or that might be nice to see explored a little more, as outlined below.

INTRODUCTION

RESULTS
- L292-297: This makes sense though it may be worth noting that regions that experience more recombination are generally likely less core/conserved so maybe naturally have higher rates of SNPs are compared to something strictly core. I don't think needs more analysis, but a sentence or two in discussion on why this (or some other explanation) might be the case would be good.
- L308-313: In light of this, do you think more 'weight' should be put on ANI vs SNPs? Can you tell if a single plasmid transfer event or something? In which case how do you weight a plasmid or MGE acquisition compared to a SNP? Not sure there is an answer to this. Both are a “single evolutionary event” but how do they compare?

DISCUSSION
- Do you know what might be causing the increase in ESBL E. coli prevalence over time? Were there any notable changes in the community/environment/screening approach that might have changed this? You do address a few things that do not change/are not different, in your results, but do you then have any theories as to whether this might be attributed to an unknown factor or simply to natural variation over time?
- Regarding the sustained blaCTX-M-15 prevalence over time (and noting its prevalence in other papers), do you hypothesise the presence of this gene is driving selection of the strains present and therefore sample? Or too much bias/not enough data to theorise?
- Do you know if the borehole water undergoes any form of treatment, and if so what impact this may have (if any) on rates of ESBL E. coli?

METHODS
- You note that contamination ranged from 0.04-1.14%; did you have an upper limit for contamination resulting in samples being excluded? If yes, can you clarify what it was and/or confirm it was above the 1.14% as no exclusions noted.
- In the Malawi_ESBL_E_coli_isolates.csv file in the github provided it says that you used Kraken for speciation but I can’t find mention of this in your methods – please add/adjust as appropriate.
- The ANI --> strains --> medoid selection by ANI per strain --> medoid as reference for SNPs within strain process is intriguing! I would like to know a little more as I have not seen this precise approach before and would like to see a little elaboration on it. For example, your work shows that ANI is more susceptible (though perhaps not to a significant extent?) to variation in accessory gene content than core SNPs might be (accounting/scaling for recombination etc). Though the ANI-defined medoid is then used as a reference for identifying core SNPs within strain. Do you think a core SNP defined medoid might have behaved differently? Similarly, do you know (or have any insight from your work here) if the ANI medoid is better for capturing both strain accessory/AMR and SNPs of the strain, than a SNP defined medoid would have been? Please note I don’t think additional work/sequencing is required, but perhaps a simple comparison of medoid selection using ANI vs SNPs (raw or scaled to account for core genome size)? Also, do you have a sense of how much impact there might be from choosing a non-medoid strain (within strain) as the reference? It may well be there is minimal impact but this would be good to know. Note: this does not necessarily require additional work and/or inclusion/discussion in the paper, but if some of these considerations look like they might have a significant impact then worth including a brief discussion on this.

FIGURES/TABLES
- Minor but for figure 1c, can I recommend trying to collapse the ‘district’, ‘year’ and ‘esbl gene’ into a single column each and removing horizontal spacing? Though more ‘squashed’, this may make it easier for the reader to trace across and see which isolate matches each of the other categories – currently, the white space makes it harder to visually track across. (Not essential, and my suggestion may also look worse, it's hard to tell without trying it – I leave it up to the authors to trial and/or decide).
- Supp figure 2: Consider drawing a line at ANI threshold and/or colouring branches to show clusters formed. Not essential but would be a nice addition to visualise more easily.

GENERAL/MULTI-SECTION
- You mention that the blaCTX-M-15 gene is highly common/conserved here, despite strain diversity and fluctuation/replacement over time. Do you think there is possible plasmid and/or other mobile genetic element sharing going

on in these populations? Could you included a comparison (even a simple alignment) or your closed genomes' with a focus on ESBL-encoding plasmids/chromosomes? Might this show any evidence the same plasmid(s) are circulating/spreading? And/or conserved structures within plasmids (other mobile genetic elements, perhaps with shared AMR gene content)?

(Remarks on code availability)
I have reviewed the code in full and re-run everything and have a couple of suggestions and errors below:

- Add a note to code that all downloaded data from the GitHub should be in a single folder called ‘Data’ for R to read it in with the current code. Also Figures generated appear to be automatically written out to a Folder called ‘Figures’ so note that this folder is needed (though can be created when first figure is generated - R offers this as a suggestion automatically when the ‘Figures’ folder can’t found found in the same path.

- Can’t run the “Phylogenetic tree with annotation” section of the code as the human_esbl_core_snps.aln.treefile code as the is not available currently on the GitHub for download and needs adding.

- When plotting the “SN_distance_violin” bit of code, there is an error because colour palette “Set4” is called but this is not a standard colour Colour Brewer palette, may be a custom one or a typo (Set1, 2 and 3 are available in Colour Brewer palettes). Note that an error regarding this is actually also captured in their html code R markdown document “## Warning: Unknown palette: "Set4"”, and doesn’t prevent the plot being plotted exactly as portrayed in their code and the relevant support figure.

- Bit of code: “tree_metadata_for_join <- tree_metadata %>%
rename(label = Isolate)” Results in error “Error: object 'Isolate' not found” meaning the ‘tree_metatdata_for_join’ is not created and the following plot using this input cannot be made. I’m not sure quite what is wrong here but I overcame this error using the following code “> tree_metadata_for_join <- tree_metadata %>%
+ tibble::rownames_to_column("label")” so I could progress through the rest. Note that same issue was seen for both the St131 and St38 trees, and both could be resolved in the same way.

Version 1:

Reviewer comments:

Reviewer #1

(Remarks to the Author)
I would like to thank the authors for the extensive work done for the revision, addressing all my queries. This is an excellent paper and I have read it with much interest.

Reviewer #2

(Remarks to the Author)
As previous, I again enjoyed reading this manuscript. The authors have carefully addressed everything highlighted in my previous review and I have no further comments or suggestions to add.

Open Access This Peer Review File is licensed under a Creative Commons Attribution 4.0 International License, which permits use, sharing, adaptation, distribution and reproduction in any medium or format, as long as you give appropriate credit to the original author(s) and the source, provide a link to the Creative Commons license, and indicate if changes were made.
In cases where reviewers are anonymous, credit should be given to 'Anonymous Referee' and the source.
The images or other third party material in this Peer Review File are included in the article's Creative Commons license, unless indicated otherwise in a credit line to the material. If material is not included in the article's Creative Commons license and your intended use is not permitted by statutory regulation or exceeds the permitted use, you will need to obtain permission directly from the copyright holder.
To view a copy of this license, visit https://creativecommons.org/licenses/by/4.0/

Thank you for taking the time to review our manuscript, titled "**Whole-genome sequencing reveals inter-household networks of gut-colonising ESBL-producing *Escherichia coli* in two rural Malawian districts**". We were pleased to hear that you are considering publishing the paper and appreciate the opportunity to revise the manuscript. We also thank the reviewers for their valuable input, which has undoubtedly helped us improve the quality of the manuscript. Below, in **bold text**, we address the reviewers' comments and outline the alterations made to the manuscript. Line numbers in our responses refer to the revised clean script, although a version with tracked changes is also available within our re-submission. Below our responses to the reviewers' comments, we also outline minor alterations made to the submitted data package to ensure compliance with *Nature Communications* policies.

<u>Reviewer 1:</u>

<u>General</u>

1.1. The manuscript reads well, presents interesting data and the methodology is sound. I especially appreciate the deep dive into the transmission network analysis including the sensitivity analysis on recombination masking. However, I have two main concerns outlined below, as well as some other major and minor queries.

Response: Thank you very much for reviewing our manuscript and for your positive comments and suggestions.

<u>Major</u>

1.2. General point 1: the discussion is missing a point in my opinion on the background prevalence of certain successful clones (such as ST10 and ST131). These lineages have extensive transmission among healthy carriers in other studies (esp. ST131). Seeing clusters ii and xii, I wonder whether these clusters represent clones normally present in the population, rather than strains being transmitted among the villages.

Response: Thank you very much for this valuable point. We further examined the SNP distances within strains belonging to successful lineages and agree that background circulation of strains from globally disseminated lineages such as ST10 and ST131 is an important consideration when interpreting genome clustering patterns. For strain xii (ST131), we show that pairwise chromosomal SNP distances within the cluster ranged from ≤10 to >25 SNPs (*Figure 2b*). Strain ii (ST10) contained genomes with pairwise SNP distances in the 10-25 range, but we note that several genome pairs within both strains remained highly similar (≤10 SNPs), always within the same village at the same timepoint, consistent with more recent local connectivity.

We have expanded our interpretation of these patterns in the Discussion (Lines 428–433): "*Several strains identified in ≥3 households belonged to globally successful lineages known to be widely disseminated across eastern Africa, including ST10, ST38 and ST131 (13). We acknowledge that some*

***clustering observed within these lineages may reflect broader background circulation and transmission from routes not studied here. However, highly similar genome pairs sharing ≤10 chromosomal SNPs that were isolated from different households within the same village at the same timepoint suggest that localised transmission also occurs*".**

Additionally, the paragraph on recommendations for inferring ESBL-Ec flow across communities now acknowledges this (Discussion, Lines 487–489): "*ANI analysis alone cannot confirm a transmission link, as strains may circulate across communities and time, requiring intense microbiological surveillance. Core genome SNP analysis remains essential for strain-specific transmission network construction*".

1.3. General point 2: Did you consider doing a risk factor analysis, to see whether any contextual data/metadata are associated with sharing a strain with another household? The manuscript is now quite ecological in nature, describing the flow of ESBL-Ec between households, but it in the current form it unfortunately offers little information which could be used to prevent ESBL-Ec transmission in a setting like this (mentioned as the first sentence in the discussion). If this was not feasible, it should at least be mentioned in the discussion.

Response: Our geospatially resolved whole-genome sequencing framework was designed to identify putative inter-household strain-sharing networks rather than epidemiological risk factors, but we agree that exploratory analysis of participant characteristics associated with carriage of strains identified in multiple households helps place strain-sharing patterns into broader epidemiological context. In the revised manuscript, we have included an exploratory analysis (Results, Lines 153–159; Supplementary Table 1): "*We explored whether participant characteristics were associated with carriage of a strain identified in multiple households during the study. After adjustment for follow-up participation, a multivariable model found no evidence that colonisation with a multi-household strain was associated with participant age, sex or district (Supplementary Table 1). Detection of multi-household strain colonisation was more likely in participants providing a follow-up sample (aOR = 2.23 [95% CI: 1.17–4.39]; P = 0.017), consistent with increased opportunity for detection across two sampling rounds*".

**Baseline WASH exposures (e.g. drinking water source and latrine use) showed very low variability within the cohort, limiting meaningful statistical inference. Although piped water infrastructure was introduced in Samama after baseline sampling, we did not originally consider reported access at follow-up to be a sufficiently reliable exposure variable because informal observations by field staff suggested continued use of communal boreholes among households with piped water access. For transparency and to enable later discussion around these issues, we have included data on this in the revised manuscript (Results, Lines 159–167): "*At baseline in 2023, no households had access to piped drinking water; however, piped water infrastructure was introduced in Samama, Mangochi District prior to follow-up sampling in 2024. At follow-up, 50 (40.7%) of 123 participants providing faecal samples in Samama reported access to piped drinking water (data were missing for one participant). However, informal observations by field staff suggested that villagers from households with piped water access continued to use communal boreholes. The prevalence of multi-household strain colonisation at follow-up among participants reporting access to piped drinking water in*

***Samama was 28.0% (95% CI: 16.7–42.7) compared to 38.9% (95% CI: 27.8–51.1) among those without access, although this difference was not statistically significant (chi-squared test: P = 0.293)*”.**

We later go on to discuss these considerations in the revised manuscript (Discussion, Lines 403–411): *“Investigation of WASH-related risk factors was limited by the near-uniformity of these exposures within the cohort. Although piped water infrastructure was introduced in Samama during follow-up, barriers to uptake that were not formally assessed in this study appeared to limit use, with boreholes remaining the predominant drinking water source. Exploratory regression analysis found no evidence that carriage of strains identified in multiple households differed by participant age, sex or district. However, our high-resolution, strain-specific genomic analyses revealed temporally and geographically localised inter-household networks of isolates sharing ≤10 SNPs, suggesting that transmission risk may be dynamic and geographically heterogeneous*”.

1.4. L140: Seventy-six ESBL-Ec were collected at baseline, but based on a prevalence of 34.1% among 211 participants, a total of 72 ESBL-Ec-positive participants are expected. Were isolates deduplicated in any way?

Response: Thank you for identifying this lack of clarity. Four samples yielded more than one morphologically distinct ESBL-Ec colony type, resulting in 76 isolates from 72 ESBL-Ec positive baseline samples being sequenced and included in the analysis. We explain this in the revised manuscript (Results, Lines 149–150). No deduplication was required, as isolates recovered from the same samples belonged to different STs and therefore represented distinct lineages.

1.5. L176-178: how does the number of virulence gene categories per strain compare to the number of virulence gene categories in strains that were detected only in a single household?

Response: In the revised manuscript, we have included details on the number of ExPEC virulence categories identified in the widespread strains with a comparison to those limited to single households (Results, Lines 208–213): “*Genes from extraintestinal pathogenic E. coli (ExPEC) virulence factor categories were detected in all 15 strains identified in ≥3 households, including those associated with adhesins in 13 (86.7%), iron uptake in 9 (60.0%), protectins in 15 (100%) and toxins in 3 (20.0%) (25). Comparable proportions were observed among the 56 strains detected in only a single household (85.7%, 66.1%, 100% and 23.3%, respectively), indicating similar distributions of major ExPEC virulence categories among strains irrespective of household distribution and network involvement*”.

1.6. L402-407: does this finding on two ST38 strains sharing <= 10 SNPs not imply that the ANI threshold was either too strict or that ANI might be somewhat inaccurate in defining strains which are later used for transmission analysis? Does this influence the recommendations in the next paragraph? How were the differences in accessory genome assessed? And did other ANI calculation methods arrive at similar measures of relatedness? I could not find information on this “outlier” isolate in the Results.

Response: Thank you for this important point regarding the 2023 ST38 network cluster identified in Mangochi District, in which one isolate (cS72c) shared <10 core genome SNPs per 5 Mb with six other

ST38 genomes but was assigned to a different ANI-defined strain. ANI- and SNP-based analyses serve complementary purposes, and we have now introduced the role of ANI-based strain identification earlier in the manuscript, before leading into core genome SNP-based transmission inference (Introduction, Lines 96–102): "*The resolution provided by whole-genome sequencing enables both bacterial strain delineation and inference of transmission networks, although these objectives require distinct analytical approaches. Isolates sharing >99.99% average nucleotide identity (ANI) are often considered to belong to the same strain and typically share >99.0% gene content (15,16). However, changes in accessory genome content may distinguish closely related strains despite high core genome similarity, while isolates belonging to the same strain are not necessarily linked by recent transmission. Fine-scale analysis of core genome variation is therefore needed to infer transmission links between isolates*".

In the revised manuscript, we have expanded our description of accessory genome divergence based on a pangenome analysis performed on this group of ST38 isolates (Results, Lines 347–353): "*Pangenome analysis revealed accessory genome divergence in the outlier isolate, cS72c. Of the 165 singleton genes identified across the cluster, 156 (94.5%) were found in cS72c, while 37 (60.1%) of the 56 genes present in six of the seven genomes were absent from cS72c. Accessory genome changes were also reflected by substantial differences in ARG profiles. Although cS72c carried the same* bla_{CTX-M} *gene as the rest of genomes in the cluster, it possessed an additional* bla_{TEM} *gene, a different dfrA allele and lacked several aminoglycoside and tetracycline ARGs present in the other isolates*". Based on the ANI-inferred strain criteria now defined in the introduction, we consider these accessory genome differences sufficient to support classification of the "outlier" isolate as a separate strain, despite close core genome similarity. Details on the use of Panaroo for the pangenome analysis have been added to the Methods (Lines 606–608).

Although we formally only use fastANI for ANI analysis in the study, for the purposes of this response, we have additionally assessed the 2023 Mangochi District ST38 genomes in question using pyani v0.2.1 (https://github.com/widdowquinn/pyani), which yielded concordant results, with the "outlier" isolate cS72c again forming its own strain based on the 99.99% ANI cutoff.

To better reflect the complementary use of ANI- and SNP-based approaches for strain identification and transmission network inference, we have refined our recommendations in the Discussion (Lines 485–489): "*same-strain genomes sharing >99.99% ANI and exhibiting high similarity across core and accessory components are likely to share a recent common ancestor when supported by epidemiological data. However, ANI analysis alone cannot confirm a transmission link, as strains may circulate across communities and time, requiring intense microbiological surveillance. Core genome SNP analysis remains essential for strain-specific transmission network construction*" … (Lines 490–493): "*Core genome SNP analysis should also be used to infer networks among closely related isolates from different ANI-defined strains, which may still be linked within networks when core genomes remain highly similar despite accessory genome variability*".

Minor

1.7. L98 and throughout the rest of the manuscript: please rephrase "clonal complexes" as this is an ambiguous term in this setting. This could for example also be interpreted as a set of related sequence types (i.e. ST-complex).

Response: We have rephrased to avoid the use of this ambiguous term here and at two further points in the revised manuscript. For example, where "clonal complexes" was first used, the revised manuscript now refers to 'transmission links' (Introduction, Lines 102–104): "*Various thresholds have been proposed to ~~identify clonal complexes~~ infer transmission links based on single nucleotide polymorphism (SNP) counts in reference-based core genome alignments of closely related isolates*".

1.8. L131-133: do you have additional data on the participants lost to follow-up? Did these differ from others based on the variables you collected?

Response: Of the 58 participants who did not provide a follow-up faecal sample, 49 remained engaged with the study team but were unable to provide a sample on the day of follow-up, rather than actively discontinuing participation. We now explain this in the revised manuscript (Results, Lines 138–140). The number of participants with whom the study team lost contact at follow-up was therefore small (7 in Mangochi District and 2 in Nsanje District). We have not included details specifically on these participants to reduce the risk of making them identifiable (as participants ending their participation or moving away may be known within the villages).

1.9. L149: were you surprised by the absence of carbapenemases in the study? This could be put into context in the discussion.

Response: Carbapenemase genes are rarely found in Malawi and carbapenems rarely used clinically, so the absence of carbapenemases in our study was not entirely surprising. This is consistent with ongoing bacteraemia surveillance at Queen Elizabeth Central Hospital in Blantyre. However, we agree that this is an important discussion point and have contextualised the finding alongside the new analysis on the genomic context of *bla*$_{CTX\text{-}M\text{-}15}$ genes that we carried out to address Reviewer Comment 2.12 (Discussion, Lines 381–385): "*The absence of carbapenemase genes was not unexpected given the limited availability of carbapenem antibiotics in Malawi and the rarity of carbapenem resistance genes reported to date (13,35,36). However, chromosomal integration of bla*$_{CTX\text{-}M\text{-}15}$ *in ESBL-Ec may facilitate future acquisition of non-ESBL-associated plasmids carrying carbapenemase genes (36–38)*".

1.10. L170-171: "we observed high levels of genome- and 171 replicon-level ARG co-occurrence (median ARG count per genome = 9)". What is meant by this? That the genomes contain many ARGs?

Response: Our intention here was to highlight that it is not just the genomes that contain various ARGs, but that ARGs often co-occur on the same replicon as the ESBL gene (e.g., on the same plasmid). We have removed the ambiguous wording in the revised manuscript (Results, Lines 194–199): "*Within the 15 strains identified in ≥3 households, from which a representative (medoid)*

***isolate underwent long-read sequencing and hybrid assembly, ~~we observed high levels of genome- and replicon-level ARG co-occurrence~~ genomes contained multiple ARGs (median count per genome = 9). ESBL genes ($bla_{CTX-M-15}$, n = 12; $bla_{CTX-M-27}$, n = 3) were mostly plasmid-borne (9/15). On these ESBL gene-harbouring plasmids of varying sizes (68.4–173.8 kbp) and incompatibility groups (5 IncF, 3 IncY and 1 IncB/O/K/Z), 2–11 other (non-ESBL) ARGs were co-located (Supplementary Table 2).*"**

1.11. L225: Do you have any additional information on the location of boreholes? Is the geographical distance a proxy measure for sharing of a borehole?

Response: We agree that shared boreholes may contribute to localised strain transmission networks involving nearby households. However, borehole locations and drinking water were not systematically sampled in the study. We now include additional information on borehole distribution in the villages and discuss the possibility that the observed network structure may reflect shared water access (Discussion, Lines 412–416): "*Boreholes in the study villages are distributed such that approximately 20 households typically share a single source within short walking distance, raising the possibility that shared drinking water access contributes to strain-sharing between nearby households. As we did not sample latrine waste or drinking water, we were not able to directly assess the involvement of WASH facilities in inter-household networks*". In the concluding paragraph of the Discussion, we also now highlight investigation of water-centred transmission pathways as an important area for future work (Lines 526–527).

1.12. L401: please add "%" after 99.99

Response: "%" has been added. Thank you for spotting this error.

1.13. L574: why were these geographical distance thresholds selected?

Response: We have clarified the rationale for the distance thresholds in the revised manuscript, which were driven by inspection of the raw data (Results, Lines 238–243): "*Same-strain pairs from hosts living >400 m apart were less common (23/190) than pairs from hosts living <400 m apart and tended to display higher chromosomal SNP distances (Fig. 2c, Supplementary Fig. 8). To examine spatial structure using stratified distance groups, pairs from households separated by <400 m were further divided into <200 m (88/190 pairs) and 200–400 m (79/190 pairs) groups, enabling comparison of immediate neighbours with households at intermediate distances*".

To preface this, we have also now included a correlation test before presenting the stratified analysis to demonstrate the relationship between SNP distances and raw geographic distances between households (Results, Lines 235–238): "*Within the 15 strains, genomic and spatial distances were compared between isolates sampled concurrently from different households in the same village, revealing a positive correlation between chromosomal SNP distance and geographic distance (Spearman's rank correlation rho = 0.24; P = 0.001)*"

Remarks on code availability

1.14. The code uploaded to Github is the html output of an RMarkdown file. Alongside this, all input contextual data/metadata has been uploaded to the repository. All figures and tables used in the manuscript can be generated by copy-pasting and running the code in the html file. Code for genomic analysis has not been uploaded, but this has also been covered in the Methods section.

Response: Thank you for reviewing the code.

Reviewer 2:

2.1. This is a well written and thorough paper, which employs a range of approaches for an in depth exploration ESBL E. coli, presented clearly and concisely such that I genuinely enjoyed reading this paper.

I had some suggestions, comments, and questions that I think may strengthen the paper and/or that might be nice to see explored a little more, as outlined below.

Response: Thank you very much for reviewing our manuscript and for the constructive comments and suggestions.

INTRODUCTION

[*No comments under this sub-section*]

RESULTS

2.2. L292-297: This makes sense though it may be worth noting that regions that experience more recombination are generally likely less core/conserved so maybe naturally have higher rates of SNPs are compared to something strictly core. I don't think needs more analysis, but a sentence or two in discussion on why this (or some other explanation) might be the case would be good.

Response: Thank you for this helpful suggestion. We agree that differences in SNP rates across genomic regions could contribute to the patterns observed following recombination masking. In the revised manuscript, we have added two sentences on this (Discussion, Lines 464–467): "*regions of the genome undergoing recombination may naturally have higher SNP rates than highly conserved core regions. Consequently, scaling SNP thresholds according to recombination-masked core genome alignment lengths may imperfectly account for the effects of recombination on network inference*".

2.3. L308-313: In light of this, do you think more 'weight' should be put on ANI vs SNPs? Can you tell if a single plasmid transfer event or something? In which case how do you weight a plasmid

or MGE acquisition compared to a SNP? Not sure there is an answer to this. Both are a "single evolutionary event" but how do they compare?

Response: Thank you for this point, which relates to points made by Reviewer 1 (Comments 1.2 and 1.6) on strain identification, network inference and the cluster of ST38 genomes identified in Mangochi District in 2023. We agree that core genome SNP differences and accessory genome changes should not necessarily be weighted against one another, because these reflect fundamentally different evolutionary processes. Consequently, we view ANI- and SNP-based analyses as complementary rather than competing approaches. ANI captures similarity across both the core and accessory genome and is therefore useful for assessing overall genomic similarity and strain identity. In contrast, core genome SNP analysis provides greater resolution for investigating recent transmission and is less affected by exchange of mobile genetic elements. As outlined in our response to Reviewer Comment 1.6, we have expanded the Introduction (Lines 96–102) to clarify the complementary roles of ANI- and SNP-based analyses.

The ST38 isolates identified in Mangochi District in 2023 provide a useful example of this, exemplifying how differences in the accessory genome can lead to reductions in ANI while having no effect on core genome SNPs (details of this are in our response to Reviewer Comment 1.6). Our analyses also highlight the greater resolution of SNPs vs ANI for transmission inference. As discussed in our response to Reviewer Comment 1.2, some widespread lineages, including ST131, contain isolates from the same strain (>99.99%) that may reflect background circulation rather than recent transmission. Core genome SNP analysis was required to distinguish isolate pairs likely linked within localised networks (≤10 SNPs) from those that were unlikely to share a recent transmission history (>25 SNPs).

These findings directly inform our recommendations in the Discussion, where we advocate for the use of both methods for related but different applications (strain identification and network construction), rather than placing more weight on one than the other (Lines 485–489): "s*same-strain genomes sharing >99.99% ANI and exhibiting high similarity across core and accessory components are likely to share a recent common ancestor when supported by epidemiological data. However, ANI analysis alone cannot confirm a transmission link, as strains may circulate across communities and time, requiring intense microbiological surveillance. Core genome SNP analysis remains essential for strain-specific transmission network construction"* … (Lines 490–493): *"Core genome SNP analysis should also be used to infer networks among closely related isolates from different ANI-defined strains, which may still be linked within networks when core genomes remain highly similar despite accessory genome variability".*

DISCUSSION

2.4. Do you know what might be causing the increase in ESBL E. coli prevalence over time? Were there any notable changes in the community/environment/screening approach that might have changed this? You do address a few things that do not change/are not different, in your results, but do you then have any theories as to whether this might be attributed to an unknown factor or simply to natural variation over time?

Response: Thank you for raising this important point. Although natural temporal variation in colonisation dynamics may have contributed, we now discuss the environmental changes that may have contributed to the change in prevalence, particularly flooding, which is common in Malawi and has been associated with contaminating drinking water (Lines 361–366): "*The increase in prevalence was unlikely to be linked to sample collection and processing methods, which remained consistent throughout the study, but environmental factors may have played a role. Regular flooding in southern Malawi in recent years may have altered transmission foci and opportunities during the study period through redistribution of faecal material to newly formed stagnant water bodies and contamination of groundwater obtained from boreholes for drinking (30,31)*".

2.5. Regarding the sustained blaCTX-M-15 prevalence over time (and noting its prevalence in other papers), do you hypothesise the presence of this gene is driving selection of the strains present and therefore sample? Or too much bias/not enough data to theorise?

Response: We agree that sustained predominance of *bla*$_{CTX-M-15}$ despite substantial strain turnover raises the possibility that this ESBL gene contributes to the persistence of successful ESBL-Ec lineages. However, we cannot directly show this from our data as we did not attempt to isolate non-ESBL-producing *E. coli*, hence we do not present a full ecological picture of *E. coli* within the gut. We have included a comment on this in the revised manuscript when discussing the widespread prevalence of *bla*$_{CTX-M-15}$ across ESBL-Ec within our study and globally (Discussion, Lines 371–375): "*The flux observed in ST and strain composition contrasts with the persistent predominance of bla*$_{CTX-M-15}$ *carriage within ESBL-Ec strains throughout the study. Although we cannot confirm that bla*$_{CTX-M-15}$ *carriage directly drove selection of ESBL-Ec strains in our study, this ESBL gene is widely prevalent across other eastern African locations (13,32), and globally (33), suggesting that it confers a selective advantage for wide dissemination across various E. coli lineages*".

2.6. Do you know if the borehole water undergoes any form of treatment, and if so what impact this may have (if any) on rates of ESBL E. coli?

Response: Boreholes in the villages are not routinely treated. During public-health emergencies such as cholera outbreaks, chlorine may be distributed across Malawi for home-based water treatment. However, we did not collect data on household water treatment, while a recent programme evaluation reports low uptake, inconsistent access to chlorine at dispensing points and often undetectable levels in treated water. We therefore do not think there is any evidence to suggest a potential effect of water treatment on ESBL-Ec prevalence or transmission dynamics.

METHODS

2.7. You note that contamination ranged from 0.04-1.14%; did you have an upper limit for contamination resulting in samples being excluded? If yes, can you clarify what it was and/or confirm it was above the 1.14% as no exclusions noted.

Response: We used the criteria developed by the Genomic Standards Consortium to ensure quality of draft genomes for inclusion. We now explicitly include the referenced criteria in the revised manuscript (Methods, Lines 586–589): "*Completeness ranged from 99.07–100.00% and*

***contamination ranged from 0.04–1.14%. All draft genomes were therefore deemed high-quality <u>(>90% completeness and <5% contamination</u>) (59) and were included in downstream analysis*".**

2.8. In the Malawi_ESBL_E_coli_isolates.csv file in the github provided it says that you used Kraken for speciation but I can't find mention of this in your methods – please add/adjust as appropriate.

Response: Thank you for pointing this out. The Kraken species IDs in the isolate metadata file were obtained from the sequencing provider. We did not formally use Kraken for speciation, as we had already confirmed species with the MALDI-TOF prior to sequencing (as described in the Methods). We have therefore removed the 'WGS Kraken ID' column from the 'Malawi_ESBL_E_coli_isolates.csv' file.

2.9. The ANI --> strains --> medoid selection by ANI per strain --> medoid as reference for SNPs within strain process is intriguing! I would like to know a little more as I have not seen this precise approach before and would like to see a little elaboration on it. For example, your work shows that ANI is more susceptible (though perhaps not to a significant extent?) to variation in accessory gene content than core SNPs might be (accounting/scaling for recombination etc). Though the ANI-defined medoid is then used as a reference for identifying core SNPs within strain. Do you think a core SNP defined medoid might have behaved differently? Similarly, do you know (or have any insight from your work here) if the ANI medoid is better for capturing both strain accessory/AMR and SNPs of the strain, than a SNP defined medoid would have been? Please note I don't think additional work/sequencing is required, but perhaps a simple comparison of medoid selection using ANI vs SNPs (raw or scaled to account for core genome size)? Also, do you have a sense of how much impact there might be from choosing a non-medoid strain (within strain) as the reference? It may well be there is minimal impact but this would be good to know. Note: this does not necessarily require additional work and/or inclusion/discussion in the paper, but if some of these considerations look like they might have a significant impact then worth including a brief discussion on this.

Response: The primary reason for identifying medoids within each strain was to select a representative isolate for long-read sequencing and hybrid assembly, thereby generating a high-quality within-strain reference genome for subsequent SNP analyses. As noted by Gorrie et al. (ref. 19), accurate SNP calling is facilitated by the use of a closely related reference genome; therefore, we generated our own within-strain references. The use of ANI-based medoid genomes as representatives of groups of closely related genomes has been described previously and is referenced in the Methods (refs. 64-66). A core genome SNP-based approach for representative isolate identification prior to strain-specific SNP analysis, while valid, may not have captured variation across the whole chromosome of a given strain.

To investigate the impact of the alternative SNP-based strategy, as suggested, we compared the ANI-defined medoids with representative isolates selected using pairwise SNP distances derived from the core genome alignment used to construct the study phylogeny. Although this alternative approach selected a different representative isolate in 11/15 strains, these differed by only 0–7 chromosomal SNPs from the corresponding ANI-defined medoids when mapped to strain-specific

references (median = 1 SNP). We therefore consider it unlikely that SNP-based methods for representative isolate selection would have materially affected downstream within-strain SNP analyses or inferred transmission network structure, provided that selected reference belonged to the same ANI-defined strain. We have added a brief description of this sensitivity analysis to the Methods (Lines 631–636) and included the comparison in new Supplementary Table 3.

FIGURES/TABLES

2.10. Minor but for figure 1c, can I recommend trying to collapse the 'district', 'year' and 'esbl gene' into a single column each and removing horizontal spacing? Though more 'squashed', this may make it easier for the reader to trace across and see which isolate matches each of the other categories – currently, the white space makes it harder to visually track across. (Not essential, and my suggestion may also look worse, it's hard to tell without trying it – I leave it up to the authors to trial and/or decide).

Response: Many thanks for this suggestion. We have collapsed the district, year and ESBL gene annotations into single columns and agree that this improves visualisation.

2.11. Supp figure 2: Consider drawing a line at ANI threshold and/or colouring branches to show clusters formed. Not essential but would be a nice addition to visualise more easily.

Response: Many thanks for this suggestion. We have added a red dashed horizontal line at the ANI threshold and have increased the height of the figure to improve visualisation of the strain clusters.

GENERAL/MULTI-SECTION

2.12. You mention that the blaCTX-M-15 gene is highly common/conserved here, despite strain diversity and fluctuation/replacement over time. Do you think there is possible plasmid and/or other mobile genetic element sharing going on in these populations? Could you included a comparison (even a simple alignment) or your closed genomes' with a focus on ESBL-encoding plasmids/chromosomes? Might this show any evidence the same plasmid(s) are circulating/spreading? And/or conserved structures within plasmids (other mobile genetic elements, perhaps with shared AMR gene content)?

Response: Thank you for raising this important point. Alongside Supplementary Table 2 (which gives detail on the replicons upon which ESBL genes were identified among hybrid-assembled representatives from strains found in ≥3 households), we now include more detail on the genomic context of *bla*$_{CTX-M-15}$ in the Results and Discussion, as well as an additional supplementary figure showing alignments of plasmids from the same incompatibility groups that share the same ESBL gene (Supplementary Figure 6).

In reference to strains identified in ≥3 households that underwent long-read sequencing and hybrid assembly, Results (Lines 196–205) now read: "*ESBL genes (bla*$_{CTX-M-15}$*, n = 12; bla*$_{CTX-M-27}$*, n = 3) were mostly plasmid-borne (9/15). On these ESBL gene-harbouring plasmids of varying sizes (68.4–173.8 kbp) and incompatibility groups (5 IncF, 3 IncY and 1 IncB/O/K/Z), 2–11 other (non-ESBL) ARGs were*

co-located (Supplementary Table 2). Comparative analysis of plasmids sharing the same ESBL gene and incompatibility group demonstrated conserved genetic contexts surrounding bla$_{CTX-M}$ genes, including recurrent associations with IS6 family transposases, although variations in overall plasmid structure were observed (Supplementary Fig. 6). Closely related plasmid backbones were detected within incompatibility groups, particularly among IncY plasmids carrying bla$_{CTX-M-15}$, although no ESBL plasmids from strains identified in ≥3 households aligned across their entirety. Among the six strains in which bla$_{CTX-M-15}$ was chromosomally encoded, the gene was consistently identified downstream of ISEcp1 (IS1380 family)".

We discuss these results and the implications of chromosomally integrated *bla$_{CTX-M-15}$* in the Discussion section (Lines 376–385): "*Almost half of the isolates in our study belonged to one of 15 strains identified in ≥3 households, 12 of which carried bla$_{CTX-M-15}$. Among these, half harboured bla$_{CTX-M-15}$-carrying plasmids spanning various incompatibility groups, consistent with heterogeneous plasmid backgrounds among ESBL-Ec in eastern Africa (13), while the remainder carried bla$_{CTX-M-15}$ downstream of ISEcp1 on the chromosome. This finding is consistent with the established role of ISEcp1 in the dissemination of bla$_{CTX-M}$ genes, where a single copy can mediate one-ended transposition of adjacent downstream genes (34). The absence of carbapenemase genes was not unexpected given the limited availability of carbapenem antibiotics in Malawi and the rarity of carbapenem resistance genes reported to date (13,35,36). However, chromosomal integration of bla$_{CTX-M-15}$ in ESBL-Ec may facilitate future acquisition of non-ESBL-associated plasmids carrying carbapenemase genes (36–38)".*

Remarks on code availability

2.13. I have reviewed the code in full and re-run everything and have a couple of suggestions and errors below:

Response: Many thanks for reviewing and running the code in full.

2.14. Add a note to code that all downloaded data from the GitHub should be in a single folder called 'Data' for R to read it in with the current code. Also Figures generated appear to be automatically written out to a Folder called 'Figures' so note that this folder is needed (though can be created when first figure is generated - R offers this as a suggestion automatically when the 'Figures' folder can't found found in the same path.

Response: We have added this information to the start of the R Markdown file to clarify the required file structure for reproducibility. Specifically, instructions now note that all downloaded data should be placed in a folder named 'data' within the working directory, and that generated figures are written to a 'figures' folder. This should ensure users have the necessary information to successfully re-run the code.

2.15. Can't run the "Phylogenetic tree with annotation" section of the code as the human_esbl_core_snps.aln.treefile code as the is not available currently on the GitHub for download and needs adding.

Response: Thank you for identifying this omission. We have now added the human_esbl_core_snps.aln.treefile file to the data repository.

2.16. When plotting the “SN_distance_violin” bit of code, there is an error because colour palette “Set4” is called but this is not a standard colour Colour Brewer palette, may be a custom one or a typo (Set1, 2 and 3 are available in Colour Brewer palettes). Note that an error regarding this is actually also captured in their html code R markdown document “## Warning: Unknown palette: "Set4"”, and doesn’t prevent the plot being plotted exactly as portrayed in their code and the relevant support figure.

Response: Thank you for identifying this issue. We have corrected the code to call a valid colour palette, removing the associated warning message.

2.17. Bit of code: “tree_metadata_for_join <- tree_metadata %>% rename(label = Isolate)” Results in error “Error: object 'Isolate' not found” meaning the ‘tree_metatdata_for_join’ is not created and the following plot using this input cannot be made. I’m not sure quite what is wrong here but I overcame this error using the following code “> tree_metadata_for_join <- tree_metadata %>% + tibble::rownames_to_column("label”)” so I could progress through the rest. Note that same issue was seen for both the St131 and St38 trees, and both could be resolved in the same way.

Response: Thank you for identifying this issue. Although we did not experience the same error, we have updated the tree annotation code as suggested to prevent similar issues for other users.

<u>Editorial Office requirements:</u>

De-identified data: We have reviewed the de-identified patient data (‘Participant_data.csv’) provided in the supplementary datasets and have removed covariates that are not directly used to generate our results (religion, tribe, marital status, education, and GPS coordinates).

Reporting summary checklist: We have provided an updated reporting summary checklist with our revised submission.

Code and software submission checklist: We ticked ‘Yes’ for the following question when submitting the manuscript: “Did you develop code that is central to the main work described in this manuscript?”, because we wanted to make our R script transparently available for review. However, the code and software submission checklist appears to relate to custom software. In our study, all bioinformatic and statistical analyses were performed using publicly available software that are referenced within the manuscript. As we did not generate our own software with custom code, we have not completed the code and software submission checklist. However, please advise us if we have interpreted this incorrectly.

Data and code availability statements: These sections are included in their own sub-sections after the Methods section, with links to the updated data and code repository.
